# An Evaluation of the Effects of Human Factors and Ergonomics on Health Care and Patient Safety Practices: A Systematic Review

**DOI:** 10.1371/journal.pone.0129948

**Published:** 2015-06-12

**Authors:** Xuanyue Mao, Pengli Jia, Longhao Zhang, Pujing Zhao, Ying Chen, Mingming Zhang

**Affiliations:** 1 Chinese Evidence-based Medicine Centre, West China Hospital, Sichuan University, Chengdu, 610041, P.R. China; 2 School of Foreign Languages, Sichuan University, Chengdu, China; University of Bremen, GERMANY

## Abstract

**Background:**

From the viewpoint of human factors and ergonomics (HFE), errors often occur because of the mismatch between the system, technique and characteristics of the human body. HFE is a scientific discipline concerned with understanding interactions between human behavior, system design and safety.

**Objective:**

To evaluate the effectiveness of HFE interventions in improving health care workers’ outcomes and patient safety and to assess the quality of the available evidence.

**Methods:**

We searched databases, including MEDLINE, EMBASE, BIOSIS Previews and the CBM (Chinese BioMedical Literature Database), for articles published from 1996 to Mar.2015. The quality assessment tool was based on the risk of bias criteria developed by the Cochrane Effective Practice and Organization of Care (EPOC) Group. The interventions of the included studies were categorized into four relevant domains, as defined by the International Ergonomics Association.

**Results:**

For this descriptive study, we identified 8, 949 studies based on our initial search. Finally, 28 studies with 3,227 participants were included. Among the 28 included studies, 20 studies were controlled studies, two of which were randomized controlled trials. The other eight studies were before/after surveys, without controls. Most of the studies were of moderate or low quality. Five broad categories of outcomes were identified in this study: 1) medical errors or patient safety, 2) health care workers’ quality of working life (e.g. reduced fatigue, discomfort, workload, pain and injury), 3) user performance (e.g., efficiency or accuracy), 4) health care workers’ attitudes towards the interventions(e.g., satisfaction and preference), and 5) economic evaluations.

**Conclusion:**

The results showed that the interventions positively affected the outcomes of health care workers. Few studies considered the financial merits of these interventions. Most of the included studies were of moderate quality. This review highlights the need for scientific and standardized guidelines regarding how HFE should be implemented in health care.

## Introduction

Many patient safety incidents are related to the lack of attention to human factors and ergonomics (HFE) in the design and implementation of technologies, processes, workflows, jobs, teams and socio technical systems [1]. Similarly, the Institute of Medicine (IOM) reported that patient safety is directly influenced by medication errors, adverse drug events, duty hours, fatigue and health care workers’ working conditions [2–4]. There is growing evidence demonstrating that human factors are the key component in adverse events [5–6]. It is inevitable that even an experienced, motivated individual with professional and undoubted skills will make a mistake in a complex health care system because, from the human factors perspective, errors usually occur when systems and technology are mismatched with human characteristics [7]. HFE is now recognized as a key discipline to help reduce or mitigate medication errors, improve the design and implementation of health information technology, and eliminate hazards that contribute to patient falls [1]. Much emphasis has recently been placed on HFE approaches to patient safety [8]. For instance, the issue of human factors was included in the World Health Organization’s (WHO) patient safety curriculum for medical students in 2009 [9]. The report from the IOM and the National Academy of Engineering lists human factors as a key systems engineering tool for designing and improving health care systems and for improving the quality of care and patient safety [10].

According to the definition of the International Ergonomics Association (IEA), “Ergonomics (or human factors) is the scientific discipline concerned with the understanding of interactions among humans and other elements of a system, and the profession that applies theory, principles, data and methods to design in order to optimize human wellbeing and overall system performance.” [11]. The general objective of HFE within the health care domain is to maximize the system’s overall performance while promoting the health, safety, comfort, and quality of the working lives of health care workers [12–13]. HFE is a core element of patient safety strategies [14]. From an HFE viewpoint, patient safety activities should reduce and mitigate medical errors, as well as improve human wellbeing, which includes job satisfaction, motivation and technology acceptance [1]. It is believed that health care workers who experience discomfort or are injured or fatigued have a higher probability of making errors that affect patient safety [15].

The use of HFE in health care and patient safety consists of three specialty domains [8]: (1) physical ergonomics, which is concerned with physical activity, including the design of medical devices, health care facilities and patient rooms that consider the physical strengths and limitations of humans; (2) cognitive ergonomics, which is concerned with mental processes, including the design of usable interfaces for health information technologies and training programs; and (3) organizational ergonomics, which is concerned with sociotechnical systems and the design of the overall work system, including work times, health care jobs and organizations that consider the fit and interactions between the different elements within the system. All these HFE domains can influence patient safety [16]. In most cases, to solve the problems of health care and patient safety, we require the application of HFE principles and methods from multiple domains.

Recently, HFE research in health care and patient safety has focused on system resilience [1, 17], or “the ability of systems to anticipate and adapt to the potential for surprise and failure” [1, 18]. Several published systematic reviews have investigated the effectiveness of ergonomic interventions in various fields, including office interventions among computer users [19], workplace ergonomic interventions with economic analyses [20] and participatory ergonomic interventions [21]. Given the growing awareness of the importance of the human factors and ergonomics in health care and patient safety, it is surprising that systematic reviews of the effects of ergonomic interventions in this field are lacking. Therefore, we sought to conduct a systematic review to evaluate the effect of HFE on health care and patient safety.

## Methods

### Inclusion Criteria

Studies were included if they satisfied the following criteria.

#### Study design

Studies with comparative designs were included, such as randomized controlled trials (RCTs), concurrent controlled studies, and before-after studies with or without controls.

#### Study subjects

Health care workers working in a health care environment (physicians, surgeons, nurses or medical students and patients).

#### Interventions

The intervention groups underwent HFE interventions, including physical ergonomics, cognitive ergonomics and organizational ergonomics. The control groups did not undergo any HFE interventions.

#### Outcomes

Data on at least one outcome must have been reported, including medical errors or patient safety, health care workers’ quality of working life (e.g., reduced fatigue, discomfort, workload, pain and injury), health care workers’ attitudes towards the interventions (e.g., satisfaction and preference), user performance (e.g., efficiency or accuracy), and economic analyses.

### Exclusion Criteria

We excluded conference proceedings, commentaries, editorials, reviews and non-comparative studies. Additionally, the exclusion criteria included HFE research studies of populations targeted at other fields, such as computer users and manufacturing workers.

### Literature Search

MEDLINE, EMBASE, BIOSIS Previews and the Chinese Biomedical Database (CBM) were searched for articles published from 1996 to Mar.2015. Relevant reference lists of all studies selected for review were manually searched for possible additional studies. The following search terms were used: human engineering, human factors, ergonomics, human factors engineering, patient safety, medical errors, medication error, adverse event, adverse drug event, workload, teamwork, fatigue, job satisfaction, working condition, occupational health, musculoskeletal diseases, worker safety, workplace stress, etc. The search terms were customized for different databases (see S1 File: Search Strategy).

### Study Selection

Two reviewers (LHZ and PJZ) independently selected studies, initially based on the inclusion criteria, the title, key words and abstract of the retrieved record were screened. When uncertainties existed, we retrieved and assessed the full text of the studies if they were available. Another reviewer (MMZ) was consulted if a consensus could not be reached.

### Quality Assessment

Two reviewers (XYM and PLJ) independently assessed the quality of all the included studies. The quality assessment tool was based on the risk of bias criteria developed by the Cochrane Effective Practice and Organization of Care (EPOC) Group [21]. This criterion was used for studies that utilized a control group, including RCTs, non-RCTs and controlled before-after studies. Studies that had before-after study designs without a control were not included in the quality assessment. We added four items [22] to assess the reporting quality and one item for the external validity assessment (Table 1). We assessed each item as “Yes” (1 point) or “No” (0 points). The quality scores were calculated and ranked on a three-category scale: poor quality (score< 6), moderate quality (score between 6 and 10), and high quality (score between 11 and 14).

**Table 1:** Quality Assessment tool.

**Reporting**
1. Was the conceptual basis of, and/or the need for the intervention explained and sound?
2. Was the intervention clearly described?
3. Were the study population and context clearly described?
4. Did the presentation and discussion of study results include all issues of concern?
**Risk of bias**
5. Was the allocation sequence adequately generated?
6. Was the allocation adequately concealed?
7. Were baseline outcome measurements similar?
8. Were baseline characteristics similar?
9. Were incomplete outcome data adequately addressed?
10. Was knowledge of the allocated interventions adequately prevented during the study?
11. Was the study adequately protected against contamination?
12. Was the study free from selective outcome reporting?
13. Was the study free from other risks of bias?
**External validity**
14. Were the subjects asked to participate in the study representative of the entire population from which they were recruited?

### Data Extraction and Synthesis

Two reviewers (XM and PLJ) independently extracted data that met the inclusion criteria using a pre-specified extraction form that included the following information: study theme and design, setting, interventions, outcome measures, measurement time, and funding. The interventions in the included studies were primarily categorized into four relevant domains, as defined by the International Ergonomics Association [11]: (1) physical ergonomics, (2) cognitive ergonomics, (3) organizational ergonomics, and (4) studies that included two or more of the previously mentioned interventions were classified as multifaceted ergonomics. Then, we systematically extracted data concerning the effectiveness of the HFE interventions on the patients, health care workers and health care system. This processed utilized five broad outcome categories: 1) medical errors or patient safety; 2) health care workers’ quality of working life; 3) user performance; 4) health care workers’ attitudes towards the interventions; and 5) economic evaluations.

## Results

### Study Searching and Selection

**Fig 1:**
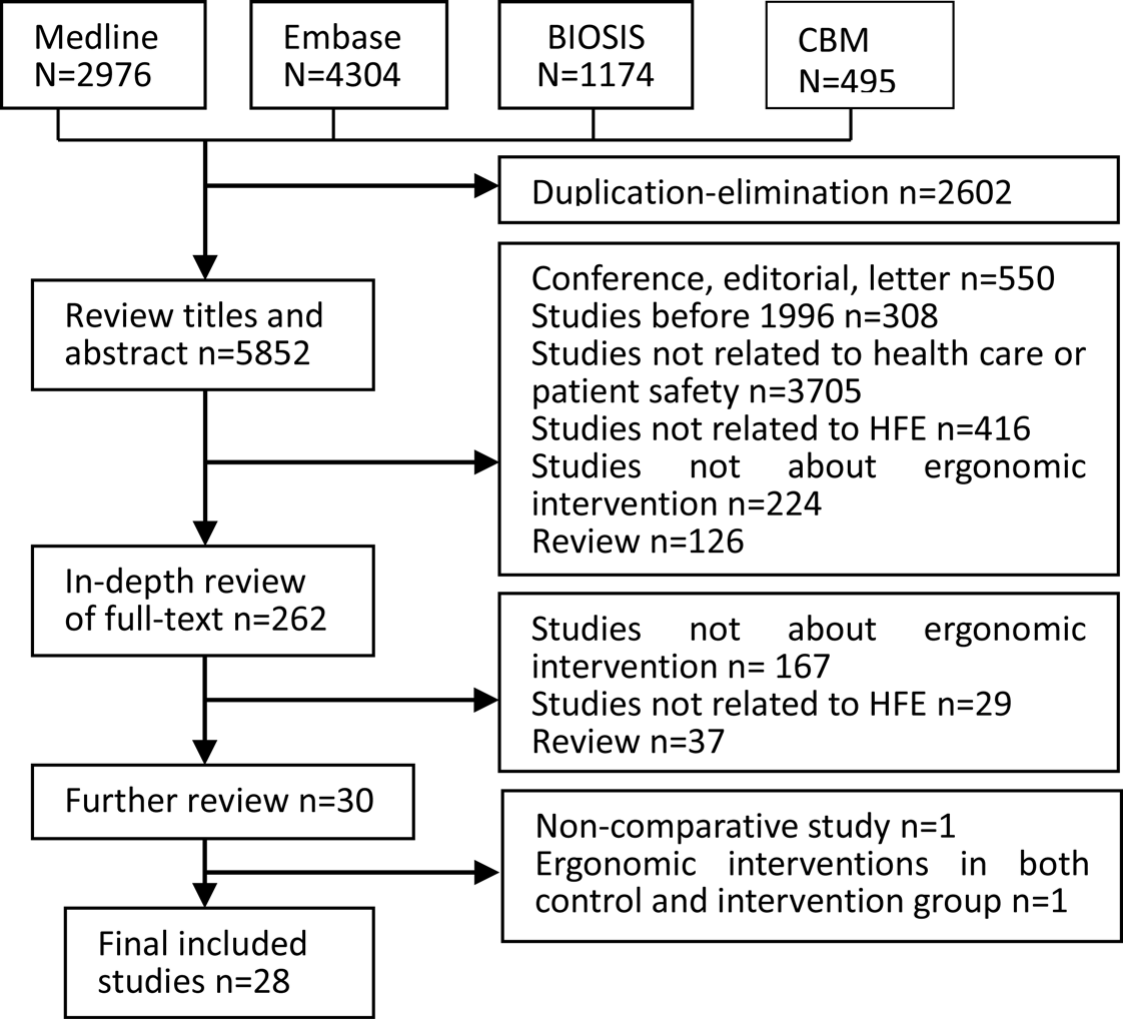
Flow Diagram for searching and selection processes.

We identified 8, 949studies based on our initial searching; Initial sifting based on title, abstract and full text resulted in exclusion of 6336 studies; 30 studies were further reviewed and two studies were excluded (non-comparative study [23], ergonomics intervention both in the control and intervention groups [24]).Finally, 28 studies with 3,227 participants were included in the final analysis (Fig 1).

### General Study Characteristics

The included studies varied in terms of the study settings, approaches to HFE interventions, and outcome measurement levels. Most of the studies were from developed countries; eleven studies were conducted in the USA [25–35], two were conducted in Canada [36–37], ten studies were conducted in Europe [38–47], two studies were conducted in Hong Kong [48–49] and three studies were conducted in Israel, Brazil and Iran [50–52]. Twenty studies were controlled studies [25–27,29,32,34,36–44,46,48,49,51,52], two of which were RCTs [44, 51]. The other eight studies were before/after surveys, without a control group [28, 30, 31, 33, 35, 45, 47, 50]. The target populations were staff working in hospitals and nursing homes, including nurses [35, 37–39, 41–43, 45, 47–49, 51], surgeons (3 studies) [27, 44, 46], medical students (4 studies) [25,32,36,52] and other health care workers (8 studies) [26, 28–30, 33–34, 40, 50]. Seventeen studies reported their funding resources [26, 28–31, 33–37, 43, 45–49, 52] and eleven were supported by public funding [26–28, 32–35,41,43–44,46]. Nine studies did not report funding source [27, 32, 38–40, 42,44–45,51].

### Characteristics of the HFE Interventions

There was substantial heterogeneity across the studies in the performance of the HFE interventions. Thirteen studies of physical ergonomic interventions [25–29, 36–37, 40, 43–44, 46, 50, 52] primarily focused on the change of working posture, the physical design of medical equipment (dentistry chairs, laparoscopic grasping tools, patient-controlled analgesia, and bag-valve mask) and workplaces. Four studies performed cognitive ergonomic interventions [30, 38–39, 51] (e.g., HFE education or training). Seven studies applied organizational ergonomics, including implementing ergonomic shift schedules for nurses and ergonomic interventions focused on the work content and clinical system design [31–33, 41–42, 45, 47]. Four studies performed multifaceted ergonomic interventions [34–35, 48–49].

### Quality Assessment

Twenty studies were included in the quality assessment. Most of the studies demonstrated good reporting quality; however, most of the studies had a high risk of bias in terms of study design, and only two studies [44, 51] performed adequate allocation sequences. The quality of the majority of the studies was moderate, with a mean score of 8.25.One study was rated as low quality, with a score of 5 [38], and one study was deemed to be of high quality, with a score of 11 [39]. The sequence generation process was adequately generated in two studies (10%) and no study allocation adequately concealed the allocation; The baseline outcomes were well measured in 11 studies (55%); The relevant outcomes presented in the methods section were reported in the results section in 19 studies; There was no evidence of other risk of biases in 13 studies. Details are reported in Table 2. The remaining eight before/after studies without controls were not included in the quality assessment.

**Table 2:** Quality of included studies.

Study ID		Reporting				Risk of bias								External validity		Score	Grading
Author	Year	1	2	3	4	5	6	7	8	9	10	11	12	13	14		
1.Szeto	2013	Y	Y	Y	Y	N	N	N	Y	N	N	Y	Y	Y	Y	9	**moderate**
2.Xiao	2012	Y	N	Y	N	N	N	Y	Y	Y	N	N	Y	Y	Y	8	**moderate**
3.Haddad	2012	Y	Y	Y	Y	N	N	N	N	Y	N	Y	Y	Y	Y	9	**moderate**
4.Lim	2011	Y	Y	Y	Y	N	N	Y	N	N	N	N	Y	Y	Y	8	**moderate**
5.Szeto	2010	Y	Y	Y	Y	N	N	N	Y	Y	Y	N	Y	N	Y	9	**moderate**
6.Bauman	2010	Y	Y	Y	Y	N	N	N	N	Y	N	Y	Y	Y	N	8	**moderate**
7.Cahan	2010	Y	Y	Y	Y	N	N	N	N	Y	N	N	Y	Y	Y	8	**moderate**
8.Park	2009	Y	Y	Y	Y	N	N	N	N	N	N	Y	Y	Y	Y	8	**moderate**
9.Galleano	2006	Y	Y	Y	N	Y	N	Y	Y	Y	N	Y	N	Y	N	8	**moderate**
10.Trejo	2006	Y	Y	Y	N	N	N	Y	N	Y	N	N	Y	N	Y	7	**moderate**
11.Smedley	2003	Y	N	Y	Y	N	N	Y	Y	Y	N	N	Y	Y	Y	9	**moderate**
12.Smith	2002	Y	Y	Y	N	N	N	Y	N	N	Y	N	Y	N	N	6	**moderate**
13.Lin	2001	Y	Y	Y	Y	N	N	Y	Y	Y	Y	N	Y	N	N	9	**moderate**
14.Alexandre	2001	Y	Y	Y	Y	Y	N	N	Y	Y	N	Y	Y	Y	N	10	**moderate**
15.Boggild	2001	Y	Y	Y	Y	N	N	Y	N	Y	Y	Y	Y	Y	N	10	**moderate**
16.Lin	1998	Y	Y	Y	Y	N	N	N	N	Y	Y	N	Y	N	N	7	**moderate**
17.Luttmann	1998	N	Y	Y	Y	N	N	N	N	Y	N	N	Y	Y	N	5	**low**
18.Engels	1998	Y	Y	Y	Y	N	N	Y	Y	Y	Y	N	Y	Y	Y	11	**high**
19.Pohjonen	1998	Y	Y	Y	Y	N	N	Y	Y	Y	N	N	Y	N	Y	9	**moderate**
20.Engels	1997	Y	N	Y	Y	N	N	Y	Y	Y	N	N	Y	N	Y	8	**moderate**
21.Rozenbaum	2013	-	-	-	-	-	-	-	-	-	-	-	-	-	-	-	**exclude**
22.Kobayashi	2013	-	-	-	-	-	-	-	-	-	-	-	-	-	-	-	**exclude**
23.Hakola	2010	-	-	-	-	-	-	-	-	-	-	-	-	-	-	-	**exclude**
24.Lavender	2007	-	-	-	-	-	-	-	-	-	-	-	-	-	-	-	**exclude**
25.Albayrak	2007	-	-	-	-	-	-	-	-	-	-	-	-	-	-	-	**exclude**
26.Nelson	2006	-	-	-	-	-	-	-	-	-	-	-	-	-	-	-	**exclude**
27.Fujishiro	2005	-	-	-	-	-	-	-	-	-	-	-	-	-	-	-	**exclude**
28.Bradley	1999	-	-	-	-	-	-	-	-	-	-	-	-	-	-	-	**exclude**

Note:—: Not applicable Y: Yes N:No.

### Effects of the Ergonomic Interventions on Health Care and Patient Safety

In this study, we defined medical errors or patient safety as the main outcome measurements; eight studies reported these outcomes [30, 35–39, 44,50]. The most common outcome was the assessment of health care workers’ quality of working life (20 studies) [26–29, 33–37, 40–46, 48–49, 51–52]. Ten studies evaluated health care workers’ attitudes towards the intervention [25, 27, 29, 35–36, 41–43, 46, 52], whereas the remaining outcomes included work performance efficiency or accuracy (10 studies) [25, 31, 32, 35–37, 39, 40, 45–46] and economic analyses (4 studies) [26, 33–35]. The effects of the HFE intervention on each of the five outcome categories are discussed below.

### Outcomes of Physical Ergonomic Interventions (Table 3)

**Table 3:** Detail outcomes of physical ergonomic intervention.

Study ID	Country	Measurement time period	Study design	Setting	Subjects (Sample)	Funding	Quality	Intervention	Outcome Measures Effects				
									1	2	3	4	5
1.Haddad 2012	Iran	NR	Controlled	Department of Industrial Engineering	Students (12)	Yes	M	HFE designed dentistry chair		↑		↑	
2.Bauman 2010	USA	NR	Controlled	Department of anesthesiology	Emergency medical technician students(6),paramedic stdents(9), respiratory therapy students(17)	No	M	HFE designed facemask			↑	→	
3.Park 2009	USA	120 months	Controlled	Nursing-home	Nursing-home workers (1028)	Yes	M	Purchase HFE equipment		↑			↑
4.Galleano2006	England	NR	Randomized control trial	NR	Surgeon (99)	NR	M	HFE armrest of simulated laparoscopic surgery	↑	↑			
5.Trejo 2006	USA	NR	Controlled	Department of surgery	Surgeon (38)	NR	M	HFE designed articulating laparoscopic		↑		↑	
6.Fujishiro2005	USA	40 months	Before/after uncontrolled	86 healthcare facilities	Health care workers (NR)	Yes	M	Purchase HFE devices		↑			
7.Smedley2003	England	33.5 months	Before/after controlled	2 hospitals	Nurses (1,239)	Yes	M	Purchase HFE equipment		↑		↑	
8.Smith 2002	USA	NR	Controlled	Department of dentistry	Novice participants (12)dental hygienists (5)	Yes	M	HFE method of viewing teeth while performing simulated dental procedures		↑		↑*	
9.Xiao 2012	Netherlands	NR	Controlled	Surgical department	Surgeons (20)	Yes	M	HFE training posture during laparoscopic surgery		↑	↑	↑	
10.Luttmann 1998	Germany	>12 months	Controlled	Urinary surgery	Urologists (15)	NR	L	HFE arrangement of the operation equipment.		↑			
11.Lin 2001	Canada	NR	Controlled	Recovery room	Nurses (12)	Yes	M	HFE interface designed PCA device	↑	↑	↑	↑	↑
12.Lin 1998	Canada	NR	Controlled	Recovery room	Nursing students (12)	Yes	M	HFE interface designed PCA device	↑	↑	↑		
13.Rozenbaum 2013	Israel	NR	Before/after uncontrolled	Hospitals wards	Hospitals wards (78)	No	M	HFE principle designed hospital wards	↑				

NR: Not Report; L: Low; M: moderate; H:high

↑: Improvement or changes in the expected direction;

→: No effect or without change or not sustained;

*:Changes in an undesired direct

1. Evaluation of errors or safety;

2. Health care workers’ quality of working life;

3. User performance evaluation;

4. Health care workers’ attitudes towards the interventions;

5. Economic evaluation

#### 1. Evaluation of errors or safety

Four studies evaluated the effects of the intervention on medical errors and patient safety [36, 37, 44, 50]. Galleano (2006) reported that ergonomically designed laparoscopic surgery armrests significantly reduced execution errors (p = 0.019) [44]. Rozenbaum (2013) attributed an improvement in reducing the risk of medication errors to the ergonomic designed medication room [50]. Lin (1998) and Lin (2001) reported a reduction of adverse drug events after using an ergonomic designed patient controlled analgesia (PCA) infusion pumps interface [36–37].

#### 2. Health Care Workers’ Quality of Working Life

Eleven studies evaluated the effect of the intervention on health care workers’ quality of working life [26–29, 36–37, 40, 43–44, 46, 52]. Two studies reported that the ergonomically designed operation equipment had a more favorable effect on electromyography (EMG) activity than the control group [40, 52]. Lin (1998) and Lin (2001) observed reduced workloads after an HFE patient-controlled analgesia (PCA) infusion pumps [36–37]. The results of four studies showed that body discomfort was reduced in the laparoscopic surgery armrests groups [27, 29, 44, 46]. Two studies found a reduction in work related musculoskeletal disorder (MSD) symptoms as a result of the ergonomic devices, which aid in patient handling and lifting [28, 43]. Park (2009) showed that MSD injury rates decreased after ergonomics courses conducted. [26].

#### 3. Evaluation of User Performance

Four studies evaluated the effect of the intervention on user’s performance [25, 36–37, 46]. Bauman (2010) showed that ergonomically designed facemasks yielded a higher tidal volume than the standard mask (361±104 mL vs. 264±163 mL) [25]. Lin (1998) found that the ergonomically designed patient controlled analgesia (PCA) interface was 15% faster than the old interface (P<0.025).[36]. Lin (2001) showed that the ergonomically designed PCA interface led to faster completion times than the old interface (p = 0.006) [37]. Xiao (2012) discovered that the task performance scores of laparoscopic surgeons who were trained under the optimal ergonomic simulation setting were higher than those of the traditional equipment during the laparoscopic surgery training. (p<0.05) [46].

#### 4. Health Care Workers’ Attitudes Towards the Interventions

Seven studies evaluated the effect of the intervention on health care workers’ attitudes towards the intervention [25, 27, 29, 36, 43, 46, 52]. Smedley (2003) showed that by using HFE designed patient-handling equipment in the wards, the “low job satisfaction” rate of nurses decreased from 35 to 26% [43]. Trejo (2006) reported that the surgeons preferred to use the ergonomically designed articulating laparoscopic prototype tool (p<0.001) [27].Bauman (2010) found that the students tended to use the traditional facemask because ergonomically designed facemasks resulted in operational difficulties (p = 0.002) [25]. Haddad (2012) reported that most dentists preferred to use an ergonomically designed chair (EDC), but the EDC had some difficulties turning the patient around in the laboratory during task simulation [52]. Lin (1998) found that all nurses preferred ergonomically designed patient controlled analgesia (PCA) interface (p<0.05) [36]. Xiao (2012) discovered that laparoscopic surgeons preferred the HFE designed operating room environment [46]. Smith (2002) demonstrated that in view of accuracy and effects, the dentists tended to use the operating mode of the HFE, but considering the comfort level, the dentists preferred the traditional operating posture [29].

#### 5. Economic evaluation

One study conducted a cost- effectiveness analysis and demonstrated that a $500 equipment purchase per nursing home worker was associated with a 21% reduction in back injuries. If the equipment was used for 10 years, the translated outcome would be a savings of $768 per person [26].

### Outcomes of Cognitive Ergonomic Interventions (Table 4)

**Table 4:** Detail outcomes of cognitive ergonomic intervention.

Study ID	Country	Measurement time period	Study design	Setting	Subjects (Sample)	Funding	Quality	Intervention	Outcome Measures Effects				
									1	2	3	4	5
1.Engels 1998	Netherlands	NR	Before-after controlled	NR	Nurses (24)	NR	H	HFE educational course	↑		→		
2.Engels 1997	Netherlands	18.5 months	Controlled	Nursing home	Nurses (24)	NR	M	HFE educational course	↑				
3.Alexandre 2001	Brazil	4 months	Randomized control trial	University hospital	Female nurses(56)	NR	M	Educational HFE program		↑			
4.Marshall 2007	USA	>24 months	Before-after uncontrolled	Five surgical facilities	Surgeons, technologists, anesthesiologists, anesthetists (688)	Yes	M	HFE team training program	↑				

NR: Not Report. L: Low M: moderate H:high

↑: Improvement or changes in the expected direction

→: No effect or without change or not sustained

1. Evaluation of errors or safety;

2. Health care workers’ quality of working life;

3. User performance evaluation;

4. Health care workers’ attitudes towards the interventions;

5. Economic evaluation

#### 1. Evaluation of Errors or Safety

Three studies reported the effects of ergonomic education projects on medical errors and patient safety [30, 38–39]. The Engels studies (1997 and 1998) showed that an ergonomic education project resulted in a decrease in the harmful postures of nurses from 37 to 17% (p<0.01) [36], and errors that conflicted with ergonomic principles decreased from 56 to 42% (p<0.01) [39]. Marshall (2007) reported that an ergonomic education project improved the awareness of patient safety and increased teamwork behavior and performance [30].

#### 2. Health Care Workers’ Quality of Working Life

One study examined the effect of an HFE intervention on health care workers’ quality of working life and showed that there was a statistically significant decrease in the frequency and intensity of cervical pain in nurses (p = 0.008) [51].

#### 3. User Performance Evaluation

One study investigated the effect of an HFE intervention on user performance and showed that the mean time spending on tasks increased after the ergonomic-educational course training compared to the control group in nurses. This result may have occurred because the ergonomic educational course changed the manner in which the trainees work, or perhaps the trainees in the intervention group were more aware of their working postures. [39]

### Outcomes of Organizational Ergonomic Interventions (Table 5)

**Table 5:** Detail outcomes of organizational ergonomic intervention.

Study ID	Country	Measurementtime period	Study design	Setting	Subjects (Sample)	Funding	Quality	Intervention	Outcome Measures Effects				
									1	2	3	4	5
1.Kobayashi 2013	USA	1.2 months	Before/after uncontrolled	Emergency department clinical systems	Emergency department clinical systems (NR)	Yes	M	HFE Improved clinical system performance			↑*		
2.Hakola 2010	Finland	12 months and 12 months follow up	Before/after uncontrolled	Hospitals	Nurses (75)	Yes	M	HFE improved shift schedules			↑		
3.Cahan 2010	USA	3 months	Controlled	Medical school	Medical students (148)	NR	M	HFE training of communication skills			↑		
4.Pasanen 2013	Denmark	12 months follow up	Before/after uncontrolled	NR	Nurses (48)	Yes	M	HFE shift schedules		↑			
5.Evanoff 1999	USA	24 months	Before/after uncontrolled	Hospital	Hospital Orderlies (NR)	Yes	M	Participatory worker–management HFE team among hospital orderlies		↑			↑
6.Boggild 2001	Denmark	12 months and 6 months follow up	Before/after controlled	Hospital wards	Nurses (101)	NR	M	HFE shift schedules		↑		→	
7.Pohjonen 1998	Finland	12 months	Before/after controlled	NR	Home care worker (70)	NR	M	HFE intervention on the work content and load		↑→	↑	↑	

NR: Not Report L: Low; M: moderate; H:high

↑: Improvement or changes in the expected direction;

→: No effect or without change or not sustained;

*:Changes in an undesired direc

1.Evaluation of errors or safety;

2. Health care workers’ quality of working life;

3. User performance evaluation;

4. Health care workers’ attitudes towards the interventions;

5. Economic evaluation.

#### 1. Health Care Workers’ Quality of Working Life

Four studies reported the effects of ergonomic interventions on health care workers’ quality of working life [33, 41, 42, 45]. Hakola (2010) found that ergonomic working time arrangements in primary health care shift work provided an effective and feasible method for improving the well-being of health care workers of all ages (p *=* 0.003–0.04) [45]. Evanoff (1999) showed that participatory worker–management ergonomics teams decreased the risks of work injury (RR = 0.50, 95% CI, 0.35–0.72), lost time because of injury (RR = 0.26, 95% CI, 0.14–0.48), and injury with three or more days of time lost (RR = 0.19, 95% CI, 0.07–0.53) [33] among health care workers involved in lifting and transferring patients. Pohjonen (1998) indicated that participatory ergonomics had positive effects on developing work content and reducing the work loads of home care workers; the proportion of postures with straight back positions increased significantly (p<0.05) from 59 to 75% in the intervention group [41]. Boggild (2001) reported that ergonomic principles provided a potential method for reducing the risk of heart disease in shift workers, but there were no improvements in sleep and blood pressure levels [42].

#### 2. User Performance Evaluations

Four studies reported the effects of ergonomic interventions on the user’s performance [31–32, 41, 45]. Hakola (2010) reported that the Work Ability Index (WAI) increased from 37.5±7 to 38.1±7 after an intervention of ergonomic shift schedules [45]. Pohjonen (1998) found that the WAI in the intervention group utilizing the ergonomic participatory approach was significantly higher compared to the control group (p<0.05), and the study population better used their abilities and requirements to adjust their work rate (p<0.05) [41]. Cahan (2010) showed that empathy scores of medical students were not significantly different in the human factors curriculum trained vs untrained groups in a pilot study with concurrent controls (p = 0.53), but the scores did improve from 2.32 to 3.45 on a 5-point scale (p<0.001) in a second pilot study with a before group and controls [32]. Kobayashi (2013) documented that the intervention of an ergonomic telemetry system improved the discovery rate of arrhythmias, but it resulted in frequent false positive alarms in the emergency department [31].

#### 3. Health Care Workers’ Attitudes Towards the Interventions

Two studies reported the effects of ergonomic interventions on the health care workers’ attitudes towards the intervention [41–42]. Pohjonen (1998) showed that 98% of the home care workers was satisfied with the participatory ergonomic intervention [40]. Boggild (2001) reported that there was no increase in job satisfaction after an ergonomic working arrangement intervention in hospital wards [42].

#### 4. Economic Evaluation

One study performed an economic evaluation. Evanoff (1999) performed a cost- effectiveness analysis and found that the workers’ compensation costs (adjusted for these temporal trends) were approximately $22.758 with the ergonomic intervention over a two-year period, but the invention cost was less than $5,000 (including the equipment and wages of the individuals conducting the intervention) [33].

### Outcomes of Multifaceted Ergonomic Interventions (Table 6)

**Table 6:** Detail outcomes of multifaceted ergonomic intervention.

Study ID	Country	Measurement time period	Study design	Setting	Subjects (Sample)	Funding	Quality	Intervention	Outcome Measures Effects				
									1	2	3	4	5
1.Nelson 2006	USA	9 months and 9 months follow up	Before/after uncontrolled	19 nursing home care units and 4 spinal cord injury units	Nurses (825)	Yes	M	An multifaceted HFE program	↑	↑	↑	↑	↑
2.Lin 2011	USA	60 months and 24 months follow up	Before/after controlled	6 different hospitals	Health care workers (1480)	Yes	M	An multifaceted HFE program		↑			↑
3.Szeto 2010	Hong Kong, China	2 months and 4 months follow up	Controlled	Community	Nurses (26)	Yes	M	An multifaceted HFE program		↑			
4.Szeto 2013	Hong Kong, China	2 months	Before/after controlled	4 public hospitals	Community nurses (50)	Yes	M	An multifaceted HFE program		↑			

NR: Not Report; L: Low; M: moderate; H:high

↑: Improvement or changes in the expected direction;

1. Evaluation of errors or safety;

2. Health care workers’ quality of working life;

3. User performance evaluation;

4. Health care workers’ attitudes towards the interventions;

5. Economic evaluation

#### 1. Evaluation of Errors or Safety

One study reported patient safety outcomes. Nelson (2006) showed the multifaceted ergonomics program intervention resulted in a statistically significant reduction in unsafe patient behavior (p = 0.027) [35].

#### 2. Health Care Workers’ Quality of Working Life

Four studies reported the effects of ergonomic interventions on healthcare workers’ quality of working life [34–35, 48, 49]. Szeto (2013) and Szeto (2010) found a significant improvement in musculoskeletal symptoms after a multifaceted ergonomic intervention programme for community nurses [48–49]. Lin (2011) and Nelson (2006) showed a reduction in the MSD injury rate with a multifaceted ergonomic intervention programme(p = 0.036) [34–35].

#### 3. Health Care Workers’ Attitudes Towards the Interventions

One study reported the effects of an ergonomic intervention on health care workers’ attitudes towards the intervention [35]. Nelson (2006) showed that work satisfaction of nurses increased after the multifaceted ergonomic intervention programme (p = 0.04) [35].

#### 4. User Performance Evaluation

One study reported the effects of an ergonomic intervention on user’s performance and showed that 96% of the nurses considered the new ergonomic equipment “very effective” [35]

#### 5. Economic evaluation

Two studies conducted economic evaluations [34–35]. Nelson (2006) performed a cost- effectiveness analysis and found that the cost of the project was $123,037, whereas there was a work related injury cost savings of $245,727 per year [35]. Lim (2011) performed a cost-effectiveness analysis and found that the work related injury cost was reduced by 41% (from $3,891 to $2,302) [34].

## Discussion

This study is the first to assess the effects of human factors and ergonomics on health care and patient safety and systematically sought evidence demonstrating the effectiveness of HFE interventions in improving outcomes of health care workers and patients. Our recent initial literature search yielded 8, 949 relevant studies, of which only 28 studies met the inclusion criteria. Most of the excluded studies were from industries that were unrelated to health care and patient safety.

### HFE Interventions in Health Care and Patient Safety

HFE research in health care covers diverse types of interventions involving a wide range of outcomes and target groups. Therefore, synthesizing the evidence was complicated by the confusion of the different types of interventions and the limited information regarding how the interventions were implemented. The results suggested that there was no evidence demonstrating which interventions were more effective than others. However, the results of two reviews concluded that ergonomic approaches that employ multiple interventions are the most successful in controlling MSD [53–54]. Approximately one-third of the studies conducted physical ergonomic interventions, whereas fewer studies conducted organizational and multifaceted ergonomic interventions. This finding may be because conducting organizational ergonomic interventions is complex and involves many aspects, such as providing a well-defined job description, redesigning work content and shifting schedules, each of which may require additional expenditure, and good outcomes may not be foreseeable in the short term. If interventions are simple, they are more likely to be adopted [55].

The human factors and ergonomic interventions fairly consistently led to improvements in both health care workers’ outcomes and patient safety [56]. Our research found that the majority of current studies (21 studies) focused on the outcome of health care workers’ quality of working life when a HFE intervention was applied, whereas few studies focused on medical errors. We did not identify any studies that explicitly measured the effect of a HFE intervention on patient safety. This major shortcoming was true for all our included studies, likely because errors are difficult to identify, and an evaluation of patient safety outcomes and medical errors are difficult to perform because there are no strict criteria used to define the medical errors or methods to detect and evaluate these errors. Consequently, most of the included studies conducted intermediate measurements of worker’s outcomes that may provide indirect evidence for reducing medical errors. It has been recognized that fewer worker injuries, better work performance and productivity, and lower worker’s compensation and hospitalization costs will result in better patient care and safety [56]. The overall results showed that the interventions had a positive effect on worker outcomes. For instance, MSD injury rates decreased after purchasing new ergonomic equipment. In addition, the economic analyses conducted by Lin demonstrated that the HFE intervention was effective in reducing staff member injuries and was also financially feasible [34]. However, in our study, the costs of HFE intervention were unclear because of the limited studies of economic evaluation, and the lack of details on data collection and calculation methods. Therefore, the financial feasibility needs to be further explored.

There is some evidence indicating that HFE interventions may have potential for improving health care for worker outcomes and seldom improving patient safety [57]. However, lack of clear patient benefit and data on harms preclude a recommendation to adopt HFE interventions for clinical practice [58]. More potent HFE interventions need to be developed and evaluated by independent researchers and primarily assess patient outcomes.

### Quality of Included Studies

This review included a wider spectrum of study designs than what is typically considered; most systematic reviews of health interventions primarily contain RCTs. Therefore, the study designs varied in quality. Additionally, the utilization of two independent reviewers and the discussion process when appraising the quality of the studies was helpful, particularly when it was difficult to determine whether a particular quality assessment item was judged as ‘Y’ or ‘N’ because of the limited information available in the published studies.

The most commonly included studies on the HFE interventions in health care and patient safety were controlled studies; therefore, the criteria developed by the Cochrane EPOC group were used to assess the risk of bias. Most of the studies met the first four items of reporting quality criteria, whereas the quality of the study design was poor. The majority of the controlled studies were of moderate quality. However, because of the nature of the intervention, RCTs would be nearly impossible to conduct in some areas, such as devices, surgical procedures and educational interventions [59–60]. Before/after studies without control groups are regarded as non-experimental designs that are commonly used in safety studies. However, from a methodological perspective, this study design detrimentally affects internal validity because we cannot be certain whether the result would have been different without the interventions [61]. However, this study design is one of the most important designs for our purposes because it is a reasonable option for evaluation and provides “a more detailed picture of our current knowledge and its limitations for clinicians and policymakers” [20].

In addition, our study found that the most of the included studies were of low or moderate quality and no obviously increasing trend in the study quality, with regard to the results of the four relevant domains(physical ergonomics, cognitive ergonomics, organizational ergonomics and multifaceted ergonomics), was visible over the years. Overall, 93% of studies were non-RCTs which were less valid research designs. The poor quality in study design may provide biased estimated effects of HFE on health care and patient safety practice. The findings also showed that the HFE had a positive effect on worker outcomes than the patient outcome. It may be because the small sample sizes and limited follow-up periods of our included studies [62–64]. Meanwhile, formal assessment for publication bias using funnel plots was not possible because the variety of outcomes and data across the studies. But it is likely that publication bias exists in this field, as shown in many others, such that the positive studies could cause overestimation the efficacy of the intervention [57]. Meanwhile the majority of studies (75%) were conducted in the US and Europe, where the nature of the clinical landscape could have affected the application and results from the HFE interventions, reducing their generalizability to other settings [65].

In conclusion, the key methodological findings from this research study are that the intervention studies presented a diversity of methodological approaches, and most of the included studies were of low or moderate quality. Similar conclusions were found in other reviews of ergonomic interventions [20, 62]. More experimental and scientific, well-designed studies are required to advance this field. To strengthen the evidence, more RCTs are required to minimize bias. Such design features are critical, although there are great challenges in conducting complex interventions, such as HFE, in the complex health care system [60].

### Strengths and Limitations of the Review

One of the key strengths of this study is its broad scope. Evidence on all aspects of the human factors and ergonomic interventions and across all sectors in health care and patient safety was considered. As in all systematic reviews, the literature search was comprehensive and thorough. In addition, detailed inclusion and exclusion criteria were developed to ensure transparency and reproducibility in the judgments. Furthermore, the utilization of two independent reviewers to perform the quality assessment and data extraction was helpful to avoid mistakes and subjective judgments.

This review has several limitations. First, there were a variety of outcomes and data across the studies; thus, a meta-analysis was impossible. Second, this review did not include gray literature because the quality of the gray literature was unknown. This exclusion may have resulted in positive publication bias because studies without statistically significant differences (in terms of effectiveness) are less likely to be published.

### Future Directions

First, larger samples and longer-term studies are required to ensure a larger and more reliable evidence base on the effects of HFE interventions on health care and patient safety. There is also a need for multiple interventions within the domain of HFE, better descriptions and reports on the implementation of the interventions, and agreement on assessment tools and metrics, all of which will help to strengthen the quality of evidence. Second, more emphasis should be placed on evaluating patient outcomes, including patient safety and medical errors. Third, additional studies must be conducted to evaluate the economics in which stakeholders are more interested. It is recommended that studies performing HFE interventions should seriously consider including an economic analysis [20].

## Conclusions

Most HFE interventions studies focus on the outcomes of health care providers instead of patient safety. Few studies have considered the financial merits of HFE. Most of the included studies were of moderate quality. This review highlights the need for scientific and standardized guidelines for implementing HFE in health care, as well as the need for HFE interventions that use more methodologically rigorous designs and multi-institutional approaches to ensure the quality of the research.

## Supporting Information

S1 Checklist PRISMA checklist. (DOC)

S1 File Search Strategy for Embase, BIOSIS Previews, Medline and Chinese Biomedical Database (CBM). (DOCX)
