# Supplementary material for: An Evaluation of the Effects of Human Factors and Ergonomics on Health Care and Patient Safety Practices: A Systematic Review
Source: PLoS One. 2015 Jun 12;10(6):e0129948. doi: 10.1371/journal.pone.0129948 (PMC4466322; doi:10.1371/journal.pone.0129948)
Supplement: S1 File — (DOCX) [file pone.0129948.s002.docx]

S1 Search Strategy.

Database: Ovid MEDLINE 1946 to Mar. 2015

Search Strategy:

--------------------------------------------------------------------------------

1 Human Engineering/ or human factor.mp.

2 human engineering.mp. or Human Engineering/

3 ergonomic.mp. or Human Engineering/

4 Human Engineering/ or human factor engineering.mp.

5 patient safety.mp. or Patient Safety/

6 medical error.mp. or Medical Errors/

7 medication error.mp. or Medication Errors/

8 Medical Errors/ or adverse event.mp.

9 adverse drug event.mp.

10 workload.mp. or Workload/

11 teamwork.mp.

12 Fatigue/ or fatigue.mp.

13 job satisfaction.mp. or Job Satisfaction/

14 Workload/ or working condition.mp. or Job Satisfaction/ or Workplace/ or Occupational

Health/

15 musculoskeletal disease.mp. or Musculoskeletal Diseases/

16 occupational health.mp. or Occupational Health/

17 Occupational Health/ or worker safety.mp.

18 workplace stress.mp.

19 1 or 2 or 3 or 4

20 5 or 6 or 7 or 8 or 9 or 10 or 11 or 12 or 13 or 14 or 15 or 16 or 17 or 18 (194619)

21 19 and 20

22 limit 21 to English language

***************************

Database: Embase <1996 to Mar. 2015>

Search Strategy:

--------------------------------------------------------------------------------

1 human engineering.mp. or bioengineering/

2 human factors research/ or human factor.mp.

3 human factor engineering.mp.

4 ergonomic.mp. or ergonomics/

5 adverse drug event.mp.

6 adverse event.mp.

7 fatigue.mp. or fatigue/ or muscle fatigue/

8 job satisfaction.mp. or job satisfaction/

9 medical error.mp. or medical error/

10 medication error.mp. or medication error/

11 musculoskeletal disease.mp. or musculoskeletal disease/

12 occupational health.mp. or occupational health/

13 patient safety.mp. or patient safety/ or health care quality/ or medical error/

14 teamwork.mp. or teamwork/

15 occupational safety/ or occupational health/ or worker safety.mp.

16 working condition.mp. or work environment/

17 workload.mp. or workload/

18 mental stress/ or workplace/ or workplace stress.mp.

19 1 or 2 or 3 or 4

20 7 or 8 or 9 or 10 or 11 or 12 or 13 or 14 or 15 or 16 or 17 or 18

21 19 and 20

22 limit 21 to English language

***************************

Database: BIOSIS Previews <1995 to Mar. 2015>

Search Strategy:

--------------------------------------------------------------------------------

1 ergonomic.mp.

2 human engineering.mp.

3 human factor engineering.mp.

4 human factor.mp.

5 adverse drug event.mp.

6 adverse event.mp.

7 medication error.mp.

8 patient safety.mp.

9 medical error.mp.

10 1 or 2 or 3 or 4

11 fatigue.mp.

12 job satisfaction.mp.

13 Occupational Health/ or occupational health.mp.

14 system.mp.

15 musculoskeletal disease.mp.

16 5 or 6 or 7 or 8 or 9 or 11 or 12 or 13 or 14 or 15

17 10 and 16

***************************

CBM <1995 to Mar. 2015>

| [**order**](app:ds:order) | **searches** |
| --- | --- |
| 1 | 缺省[智能]:人体工程学 |
| 2 | 缺省[智能]:人类工程学 |
| 3 | 缺省[智能]:工效学 |
| 4 | 缺省[智能]:人因工程学 |
| 5 | 缺省[智能]:人机工程学 |
| 6 | 缺省[智能]:人间工学 |
| 7 | #1 or #2 or #3 or #4 or #5 or #6 |
